# Running Performance at High Running Velocities Is Impaired but V′O_2max_ and Peripheral Endothelial Function Are Preserved in IL-6^−/−^ Mice

**DOI:** 10.1371/journal.pone.0088333

**Published:** 2014-02-12

**Authors:** Marta Wojewoda, Katarzyna Kmiecik, Renée Ventura-Clapier, Dominique Fortin, Marta Onopiuk, Justyna Jakubczyk, Barbara Sitek, Andrzej Fedorowicz, Joanna Majerczak, Karol Kaminski, Stefan Chlopicki, Jerzy Andrzej Zoladz

**Affiliations:** 1 Jagiellonian Centre for Experimental Therapeutics (JCET), Jagiellonian University, Krakow, Poland; 2 Department of Muscle Physiology, University School of Physical Education, Krakow, Poland; 3 U-769, INSERM, Université Paris-Sud, Châtenay-Malabry, France; 4 Department of Cardiology, Medical University of Bialystok, Bialystok, Poland; 5 Department of Biochemistry, Nencki Institute of Experimental Biology, Warsaw, Poland; Mayo Clinic, United States of America

## Abstract

It has been reported that IL-6 knockout mice (IL-6^−/−^) possess lower endurance capacity than wild type mice (WT), however the underlying mechanism is poorly understood. The aim of the present work was to examine whether reduced endurance running capacity in IL-6^−/−^ mice is linked to impaired maximal oxygen uptake (V′O_2max_), decreased glucose tolerance, endothelial dysfunction or other mechanisms. Maximal running velocity during incremental running to exhaustion was significantly lower in IL-6^−/−^ mice than in WT mice (13.00±0.97 m^.^min^−1^ *vs.* 16.89±1.15 m^.^min^−1^, P<0.02, respectively). Moreover, the time to exhaustion during running at 12 m^.^min^−1^ in IL-6^−/−^ mice was significantly shorter (P<0.05) than in WT mice. V′O_2max_ in IL-6^−/−^ (n = 20) amounting to 108.3±2.8 ml^.^kg^−1.^min^−1^ was similar as in WT mice (n = 22) amounting to 113.0±1.8 ml^.^kg^−1.^min^−1^, (P = 0.16). No difference in maximal COX activity between the IL-6^−/−^ and WT mice in m. soleus and m. gastrocnemius was found. Moreover, no impairment of peripheral endothelial function or glucose tolerance was found in IL-6^−/−^ mice. Surprisingly, plasma lactate concentration during running at 8 m^.^min^−1^ as well at maximal running velocity in IL-6^−/−^ mice was significantly lower (P<0.01) than in WT mice. Interestingly, IL-6^−/−^ mice displayed important adaptive mechanisms including significantly lower oxygen cost of running at a given speed accompanied by lower expression of sarcoplasmic reticulum Ca^2+^-ATPase and lower plasma lactate concentrations during running at submaximal and maximal running velocities. In conclusion, impaired endurance running capacity in IL-6^−/−^ mice could not be explained by reduced V′O_2max_, endothelial dysfunction or impaired muscle oxidative capacity. Therefore, our results indicate that IL-6 cannot be regarded as a major regulator of exercise capacity but rather as a modulator of endurance performance. Furthermore, we identified important compensatory mechanism limiting reduced exercise performance in IL-6^−/−^ mice.

## Introduction

It is widely recognized that interleukins are secreted by macrophages and lymphocytes to coordinate the response of the immune system to injury and infection [1]. Although interleukin-6 (IL-6) is known as a key modulator of this response [2], it is also considered as an “exercise factor” released by contracting skeletal muscles into the circulation which adjusts substrate (lipid and carbohydrate) metabolism to increased energy demand during exercise [3], [4]. Depending on the exercise intensity and its duration, plasma level of IL-6 in humans can be elevated even up to 100-fold after long lasting exercise (e.g. marathon run) and drops to its basal soon after the exercise is terminated [5]. IL-6 exerts its effects both on the whole body and skeletal muscles via endocrine and paracrine/autocrine manners, respectively, without activating classical pro-inflammatory pathways [6]. Namely, it has been postulated that IL-6 stimulates hepatic glucose release and lipolysis in adipose tissue as well as glucose uptake and fatty acid oxidation in skeletal muscles thus providing contracting skeletal muscle fibres with energetic substrates [4]. Since IL-6 is considered to be vital for regulation of glucose and lipid metabolism during exercise, its deficiency should lead to the impairment of exercise performance. Indeed, it was consistently reported that IL-6 knockout mice displayed compromised treadmill and swimming exercise capacity as evidenced by their reduced endurance time [7]–[9]. However, the data concerning regulation of the whole-body glucose and lipid metabolism in IL-6^−/−^ animals are not so evident since one group referred to age-related insulin resistance and weight gain of IL-6^−/−^ mice [10], [11] while the other did not confirm this observation [12], [13]. Therefore, these data cannot explain the reduced exercise performance of IL-6^−/−^ mice. It seems that, despite previous works, the mechanisms underlying impairment of exercise tolerance in IL-6^−/−^ animals are still not known.

It has been also reported that, in spite of lower exercise tolerance of the IL-6^−/−^ mice, their oxygen cost of running during exercise at submaximal intensities is significantly lower than in WT mice for unknown reason [7]. Fäldt *et al.* [7] postulated that it could be due to progressive oxygen depletion in these animals. If indeed it would be the case, the maximal oxygen uptake (V′O_2max_) in IL-6^−/−^ mice should be significantly lower than in the WT mice. In the present study we have hypothesized that lower exercise tolerance in the IL-6^−/−^ mice will be accompanied by lower V′O_2max_. We also examined whether lower exercise tolerance in IL-6^−/−^ could be linked to alterations in glucose tolerance, endothelial function or changes in locomotor muscle profile including activities of mitochondrial enzymes or expression of sarcoplasmic reticulum Ca^2+^-ATPase (SERCA) and uncoupling protein-3 (UCP-3).

## Materials and Methods

### Animals

The experiment protocol was approved by the Bioethics Committee of Institute of Pharmacology, Polish Academy of Sciences, Krakow, Poland (Permit Number: 914/2012). All experiments including treadmill exercise were preceded with acclimatization time to minimize stress of animals whereas surgery procedures were carried out under ketamine/xylazine anesthesia.

Eighty nine male IL-6^−/−^ mice and their age-matched wild type IL^+/+^ (WT) littermates used as controls in all experiments were bred in Medical University of Bialystok, Poland. The animals were between 7 and 10-month-old once they were enrolled into the study and since then they were housed in single cages. They were maintained on a 12∶12 h light-dark cycle and were given unlimited access to food and water for the duration of the experiment. At the end of the experiment, lack of functional IL-6 gene was confirmed by genotyping of liver samples. Namely, genomic DNA was isolated with Genomic Mini kit (A&A Biotechnology, Gdansk, Poland) and PCR was performed using DreamTaq Green PCR Master Mix (ThermoScientific, Rockford, IL) and specific primers (M_IL-6_Fwd 5′-CCATCCAGTTGCCTTCTTG-3 and M_Il-6_Rev 5′-AAGTGCATCATCGTTGTTCATAC-3′). Subsequently, DNA was separated by electrophoresis on the agarose gel with ethidium bromide and liver samples of IL-6 knockouts were distinguished from WT control animals on the basis of their size (2400 bp for IL-6^−/−^ and 1476 bp for WT mice).

### Exercise capacity and endurance training protocols

WT and IL-6^−/−^ mice were acclimatized to the motorized treadmills (closed one-lane treadmill for whole body gas exchange measurements and six-lane treadmill for the assessment of running performance capacities) (Columbus Instruments, Columbus, OH, USA) for three weeks. Subsequently, we assessed exercise capacity of both WT control as well as IL-6^−/−^ knockout animals by measuring their (1) maximal oxygen consumption (V′O_2max_) during maximal incremental test, (2) oxygen consumption (V′O_2_) at maximal running velocity (v_max_) (V′O_2_ at v_max_) and (3) endurance time (the duration of run until exhaustion at the given speed). For estimation of the V′O_2_ during incremental test, each mouse was placed on the closed treadmill at 0° incline equipped with oxygen and carbon dioxide sensors to measure the concentration of the gases in the outflowing air using the Columbus Instruments' Comprehensive Lab Animal Monitoring System (CLAMS, Columbus Instruments, Columbus, OH, USA). Once the animal was acclimatized to the treadmill and its oxygen consumption became stable, the treadmill was started at 5 m^.^min^−1^ and the speed was incrementally increased by 4 m^.^min^−1^ every 3 min until the mouse reached exhaustion defined as being unable to continue running for at least 5 s in spite of the electric stimulus. To minimize effects of any errors resulting with poor repeatability of forced exercise endurance tests for the particular rodent subjects reported by Knab et al. [14], each mouse performed this test twice with one-week interval in between and the higher V′O_2max_/v_max_ values of the two obtained were considered as the “true” V′O_2max_/v_max_ for the particular animal. Endurance time was assessed on the six-lane treadmill at the 0° incline at running velocities of 10 and 12 m ^.^ min^−1^ constituting, respectively, ∼77% and ∼95% of v_max_ of IL-6 knockout mice. After acclimatization to the treadmill and warm up at 5 m^.^min^−1^, animals were forced to run at 10 m^.^min^−1^ until exhaustion (defined as above) but no longer than 2 hrs. For estimation of their endurance time at 12 m^.^min^−1^, they warmed up at 5 and 10 m^.^min^−1^ and then were forced to run at 12 m^.^min^−1^ until exhaustion but not longer than 1 h. It is worth mentioning that both groups of mice were kept in the same conditions (same size of cages) through their life, thus the IL-6^−/−^ mice and WT mice were similarly exposed to physical activity. Therefore, any differences among them concerning their physical capacity could not be attributable to the differences in their training background but only to the presence or lack of IL-6-dependent activity.

### Monitoring of the oxygen consumption

For estimation of basal oxygen consumption (basal V′O_2_) and V′O_2_ during sub-maximal exercise, mice were acclimatized to the closed metabolic cage or closed one-lane treadmill equipped with oxygen and carbon dioxide sensors (Columbus Instruments, Columbus, OH, USA) for a week. The basal oxygen consumption (basal V′O_2_) was measured at a set time of the day (7–9 a.m.) for 1 h following 30 min of an animal acclimatization. To determine their V′O_2_ during sub-maximal exercise, mice were run at 6 m^.^min^−1^ for 1 hour.

### Temperature measurements

Body temperature at rest was assessed rectally around 9 a.m. after measurement of basal V′O_2_ (see above) whereas body temperature after sub-maximal exercise was measured before and immediately after animals completed 1-hour run at 8 m^.^min^−1^ around 9 a.m.

### Glucose tolerance test

Glucose tolerance was assessed in mice at the age of 10 and 12 months. Mice were starved for 18 hrs and, then their basal glucose concentration was measured in blood samples obtained from the tail vein and diluted with saline (ABX Pentra 400 biochemical analyser, Horiba ABX, France). Subsequently, mice were intraperitoneally injected with glucose (2 g^.^kg^−1^) and glucose concentration was measured at 15, 30, 45, 60 and 120 min time points.

### Tissue samples collection

Mice were anesthetized with ketamine and xylazine (100 and 10 mg^.^kg^−1^, respectively). Subsequently, aorta, liver and skeletal muscles (*soleus* and *gastrocnemius*) were harvested and snap-frozen in liquid nitrogen for further analysis except from aorta that was placed on Krebs buffer (118.06 mM NaCl, 4.69 mM KCl, 1.19 mM KH_2_PO_4_, 1.16 mM MgSO_4_, 2.52 mM CaCl_2_, 25 mM NaHCO_3_, 10 mM glucose, 2 mM pyruvic acid sodium, 0.030 mM EDTA), cleaned from the connective and adventitia tissues and cut into 2-mm rings. Aortic rings were subsequently mounted in Multi Wire Myograph System (620M, DMT, Denmark) and maintained in KH buffer at 37°C equilibrated with 95%O_2_-5%CO_2_ to measure acetylcholine (ACh)- and sodium nitroprusside (SNP)-dependent endothelial function. For determination of post-exercise plasma lactate concentration, separate groups of IL-6^−/−^ (n = 22) and WT animals (n = 22) were subjected to either a single bout of submaximal exercise (8 m^.^min^−1^ for 1 hour) or incremental test (as described above) and just after the run were sacrificed and their blood samples were collected. After centrifugation, plasma lactate concentration was determined with Stat Profile pHOx (Nova Biomedical, Waltham, MA).

### The blood variables and plasma lipid profile

Blood samples were collected in tubes containing EDTA and were used either to perform the blood count (animal blood counter Vet abc, Horiba Medical, France) or centrifuged to obtain plasma which was further aliquoted and stored at −80°C for determination of lipid profile at ABX Pentra 400 biochemical analyzer (Horiba ABX, France).

### Cytochrome C oxidase (COX) and citrate synthase (CS) activities

Skeletal muscle samples (*soleus* and mixed *gastrocnemius*) were homogenized in the buffer containing 5 mM HEPES, 1 mM EGTA, 0.1% Triton X-100, 1 mM DTT and 10 N KOH. COX activity was measured as a time-course increase in absorbance at 550 nm due to oxidation of its substrate 1 mM reduced cytochrome C from horse (Sigma Aldrich, St. Louis, MO) according to the equation: reduced Cyt C+O_2_→oxidized Cyt C+H_2_O. CS activity was determined as the increase of absorbance at 412 nm according to reactions: acetylCoA (0.3 mM)+oxalacetic acid (0.5 mM)+H_2_O→citrate acid+CoASH+DTNB (0.1 M)→CoAS+H+ +C_6_O_4_S^2−^ (all reagents were from Sigma Aldrich, St. Louis, MO). Subsequently, enzyme activities were normalized to protein concentration in the samples (measured with BCA assay) and the values were used to calculate COX and CS activities.

### Western blot

Skeletal muscles of non-exercising mice were homogenized in the buffer containing protease and phosphatase inhibitors (Invitrogen Corp., Camarillo, CA). The protein concentration was determined by BCA Protein Assay Kit (Thermo Scientific, Rockford, IL). Subsequently, equal amounts of protein were separated by SDS-PAGE, transferred to nitrocellulose membrane and blocked with 5% non-fat dry milk/TBS-0.05% Tween solution. The washed blots were incubated with primary antibodies at following concentrations: anti-UCP3 (1 ug^.^ml^−1^, ab3477), anti-SERCA1 ATPase (1∶1000, ab2819), anti-GAPDH (1∶10 000, ab8245). Upon washing in TBS-0.05% Tween, the blots were incubated with goat anti-mouse polyclonal secondary antibodies labelled with horseradish peroxidase (1∶5000, ab6789). Both the primary and secondary antibodies were from Abcam (Cambridge, UK). The signal was detected with chemiluminescence detection kit (Bio-Rad Laboratories, Munich, Germany).

### Statistical analysis

Statistical analysis was performed in GraphPad Prism5, Statistica 10 and MS Excel 2003. P values lower than 0.05 were considered significant. The details regarding the specific statistical tests used are presented below relevant figures.

## Results

### Running performance during maximal incremental exercise test in IL-6^+/+^ (WT) control and IL-6 knockout (IL-6^−/−^) mice

**Figure 1:**
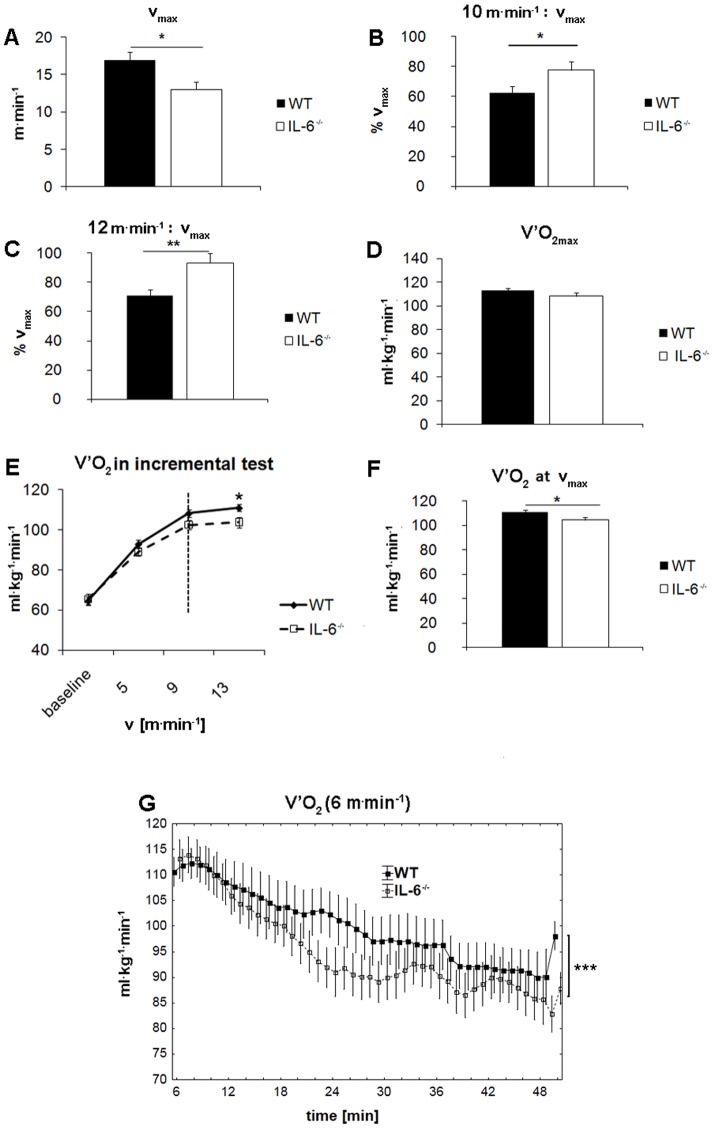
Running performance during maximal incremental exercise test in WT and IL-6^−/−^ mice. Maximal velocity (v_max_) (A), running intensity at 10 m^.^min^−1^ (B) and at 12 m^.^min^−1^ (C), maximal oxygen consumption (V′O_2max_) (D), oxygen consumption during an incremental test with increasing speeds (E), oxygen consumption at maximal velocity (V′O_2_ at v_max_) (F) and oxygen consumption during 1-hour run at submaximal velocity of 6 m^.^min^−1^ (G). For determination of v_max_ (A), V′O_2max_ (D) and V′O_2_ at v_max_ (F), WT control and IL-6^−/−^ mice were run at an inclination of 0° with the increasing speed and their oxygen consumption (V′O_2_) was registered (E) whereas for measurement of V′O_2_ during sub-maximal exercise, mice were run at velocity of 6 m^.^min^−1^ for 1 h (G). Data are presented as the mean ± SEM. The symbols * denote values significantly different: *(P<0.05), **(P<0.01), ***(P<0.001). Statistical analysis was performed in Statistica 10 (G; ANCOVA, n = 6, P<0.0001) or GraphPad Prism5 (two-sided T-test; n = 22-11).

IL-6^−/−^ and WT mice were subjected to maximal incremental exercise treadmill test to assess their maximal running velocity in this protocol (v_max_) which was by about 23% lower for IL-6 knockouts (13.00±0.97 m^.^min^−1^, n = 12) when compared with WT controls (16.9±1.2 m.min^−1^, n = 12); P<0.02) (Figure 1A). Therefore, running exercise performed at absolute intensities determined by velocities of 10 and 12 m^.^min^−1^ was more intense for IL-6^−/−^ than WT mice (Figures 1B and 1C). Simultaneously, we also monitored oxygen consumption (V′O_2_) (Figure 1E) and found that, in spite of no difference in maximal oxygen uptake (V′O_2max_) between IL-6 knockouts (108.3±2.8 ml^.^kg^−1.^min^−1^, n = 20) and WT mice (113.0±1.8 ml^.^kg^−1.^min^−1^, n = 22; P = 0.16) (Figure 1D), V′O_2_ of IL-6^−/−^ animals at v_max_ was lower (104.5±2.4 ml^.^kg^−1.^min^−1^, n = 21) when compared with WT mice 111.0±1.7 ml^.^kg^−1.^min^−1^, n = 22; P<0.04) (Figure 1F). When running at sub-maximal velocity of 6 m^.^min^−1^ for 1 h, IL-6^−/−^ mice also displayed lower V′O_2_ when compared with WT control mice (P<0.0001, F = 32.29, n = 6) (Figure 1G).

### Running performance during prolonged exercise in IL-6^+/+^ (WT) control and IL-6 knockout (IL-6^−/−^) mice

**Figure 2:**
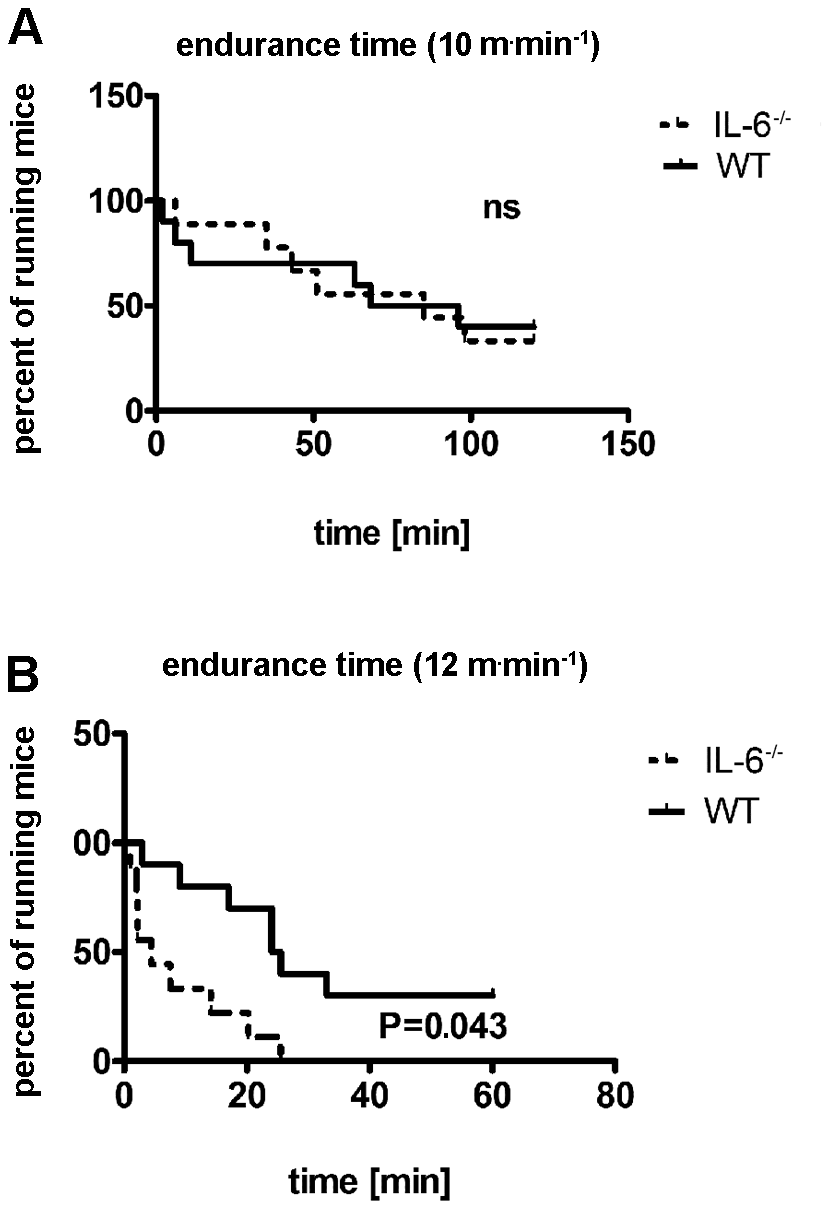
Endurance time of WT mice and IL-6^−/−^ mice. WT age-matched littermates (–) and IL-6^−/−^ mice (–) were run on the treadmill at an inclination of 0° at either 10 (A) or 12 m^.^min^−1^ (B) for 2 hrs or 1 h, respectively, or until exhaustion as described in *Materials and Methods*. The results were analyzed by the log-rank Mantel-Cox test in GraphPad Prism 5 and only P values lower than 0.05 were considered significant (n = 9–10).

It was previously reported that IL-6^−/−^ mice displayed reduced endurance time when compared with control animals [7]–[9]. Our IL-6^−/−^ mice forced to run for 2 hours at 10 m^.^min^−1^ performed as well as their WT littermates (Figure 2A) though this exercise was more intense for them due to their lower maximal velocity (Figure 1A). However, increasing the speed up to 12 m^.^min^−1^ revealed compromised endurance time of IL-6 knockout animals (Figure 2B). The discrepancy between these two speeds originated from the fact that IL-6^−/−^ mice during running at 12 m^.^min^−1^ were actually exercising close to their maximal velocity (13±1 m^.^min^−1^; Figure 1A) whereas the run at 10 m^.^min^−1^ constituted only about 77% of their maximal running velocity (v_max_) established for each mouse during the incremental running test (see Figure 1B).

### Basal V′O_2_, blood count/plasma lipid profile, body weight and temperature as well as glucose tolerance and endothelial function of IL-6^+/+^ (WT) control and IL-6 knockout (IL-6^−/−^) mice

**Figure 3:**
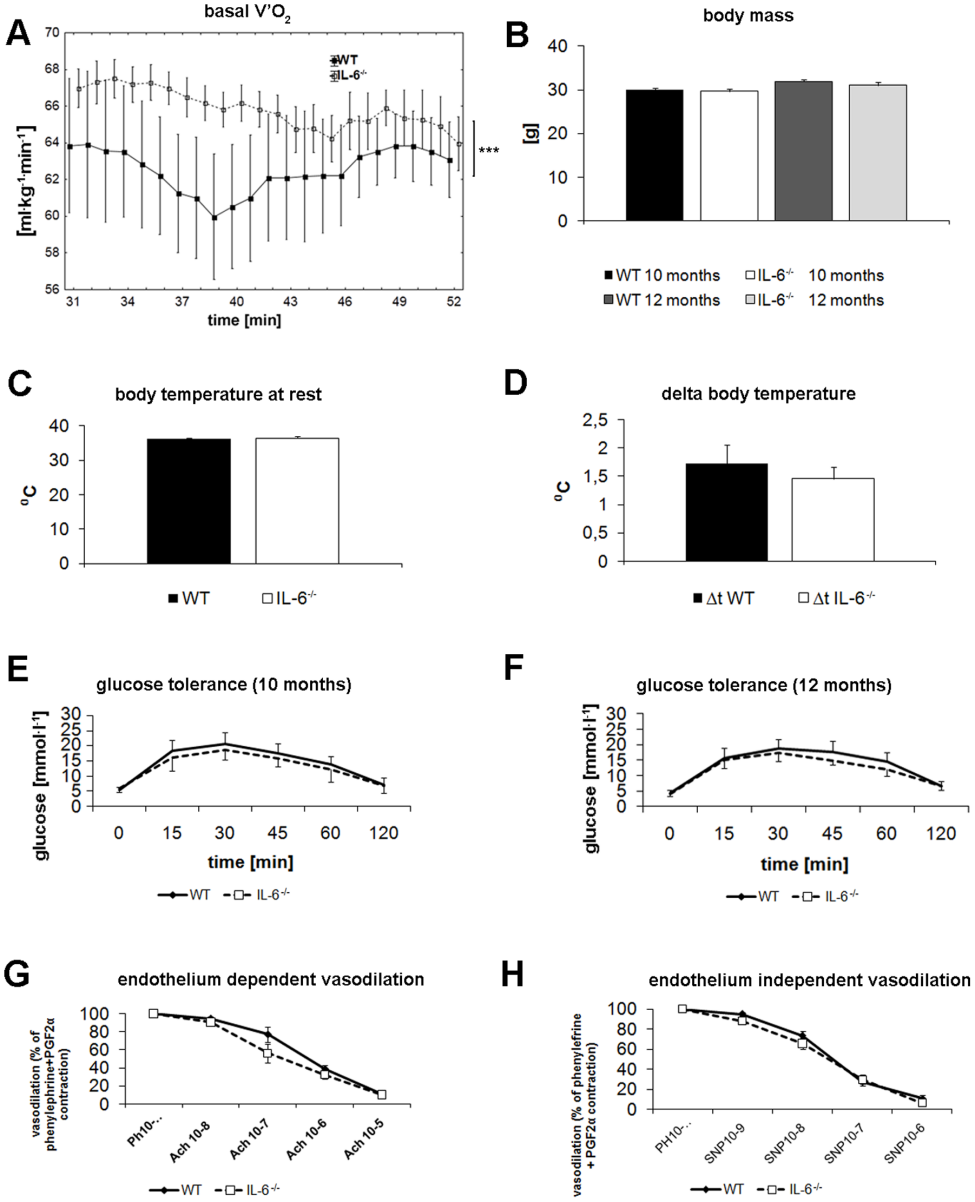
Basal V′O_2_, body weight, temperature, glucose tolerance and endothelial function of WT and IL-6^−/−^ mice. Basal oxygen consumption (A), body weight (B), resting body temperature (C), increase in body temperature after exercise (D), glucose tolerance of 10 month old mice (E), glucose tolerance of 12 month old mice (F), endothelium- dependent vasodilation of aortic rings (G), endothelium- independent vasodilation of aortic rings (H). (A) Basal oxygen consumption was measured as described in *Materials and Methods*. Glucose tolerance (E,F) of WT and IL-6^−/−^ mice at 10 and 12 month of age were compared. Temperature at rest was measured around 9 a.m. for all animals (C). Subsequently, the increase in body temperature after 1 hr run at 8 m^.^min^−1^ of WT and IL-6^−/−^ mice was compared (D). Peripheral endothelial function was assessed by the measurements of acetylcholine (Ach)- induced vasodilation (G). Subsequently, endothelium-independent SNP-induced vasodilation of the same aortic rings was record for comparison (H). Data are presented as the mean ± SEM. Statistical analysis was performed in Statistica 10 (A; ANCOVA, n = 10, P<0.0001) and in GraphPad Prism5 (two-sided T-test; n = 22–23 (B), n = 8–9 (C,D), n = 10–12 (E,F), n = 5 (G,H)).

To answer why IL-6^−/−^ mice displayed reduced endurance performance, we investigated the number of parameters that could determine their exercise capacity. Contrary to V′O_2_ during exercise, basal (resting) V′O_2_ of IL-6^−/−^ mice was significantly higher in comparison with WT control mice (P<0.0001, n = 10) (Figure 3A), indicating their increased basal metabolism. The blood count and plasma lipid profile were not different between IL-6^−/−^ and WT animals (Table 1). As it was reported that IL-6^−/−^ mice developed age-related obesity that might contribute to their reduced exercise performance [7], [11], [15], we monitored their body weight and found no difference (P>0.05) between IL-6^−/−^ and WT mice both at the age of 10 and 12 months. Since reduced exercise performance of IL-6^−/−^ mice could also result from increased energy dissipation, we measured their body temperature which was not different both at rest (Figure 3C) and just after exercise (Figure 3D) between IL-6^−/−^ and WT mice. Moreover, our IL-6^−/−^ mice also did not display an impaired glucose tolerance either at 10 (Figure 3E) or 12 months of age (Figure 3F). Subsequently, we investigated vasodilation response to acetylcholine (ACh) (eNOS dependent) and sodium nitroprusside (SNP) (eNOS independent) of pre-constricted isolated aortic rings of IL-6^−/−^ and WT control animals (Figure 3G and 3H, respectively) and found no impairment in ACh-induced vasodilation in IL-6^−/−^ vs WT mice (with SNP used as endothelium- independent vasodilation control).

**Table 1:** The blood count and plasma lipid profile of WT control and IL-6^−/−^ mice.

	WT	IL-6^−/−^
WBC [K^.^ ul^−1^]	3.03±1.33	3.23±1.36
LYM% [%]	72.5±10.7	77.74±7.61
MON% [%]	5.1±0.91	4.59±1.03
GRA% [%]	22.4±10.14	17.67±6.86
LYM [K^.^ul^−1^]	2.14±0.84	2.5±1.1
MON [K^.^ul^−1^]	0.12±0.08	0.21±0.29
GRA [K^.^ul^−1^]	0.85±0.65	0.63±0.31
RBC [M^.^ul^−1^]	9.35±0.76	9.78±0.56
HGB [g^.^dl^−1^]	13.17±0.96	13.62±0.53
HCT [%]	47.21±4.22	48.46±2.1
MCV [fl]	50.4±1.58	49.6±1.1
MCH [pg]	14.12±0.51	13.94±0.66
MCHC [g^.^dl^−1^]	27.96±1.41	28.13±1.1
RDW [%]	13.76±0.79	13.5±0.37
PLT [K^.^ul^−1^]	1561.33±223.44	1363.8±198.31
MPV [fl]	5.34±0.21	5.21±0.1
LDL [mmol^.^l^−1^]	0.0975±0.05	0.092±0.27
HDL [mmol^.^l^−1^]	0.83±0.19	1.02±0.27
TC [mmol^.^l^−1^]	1.49±0.29	1.775±0.46
TG [mmol^.^l^−1^]	0.41±0.18	0.49±0.26

WBC (white blood cells: LYM% (% of lymphocytes), MON% (% of monocytes), GRA% (% of granulocytes)), RBC (red blood cells), HGB (hemoglobin), HCT (hematocrit), MCV ((red cell) mean corpuscular volume), MCH ((red cell) mean corpuscular hemoglobin), MCHC ((red cell) mean corpuscular hemoglobin concentration), RDW (red cell distribution width), PLT (platelets), MPV (mean platelets volume), LDL (low-density lipoprotein), HDL (high-density lipoprotein), TC (total cholesterol), TG (triglicerydes). Data are presented as means ± SEM.

### Cytochrome C oxidase (COX) and citrate synthase (CS) activities in *soleus* and gastrocnemius skeletal muscles as well as post-exercise plasma lactate in IL-6^+/+^ (WT) control and IL-6 knockout (IL-6^−/−^) mice

**Figure 4:**
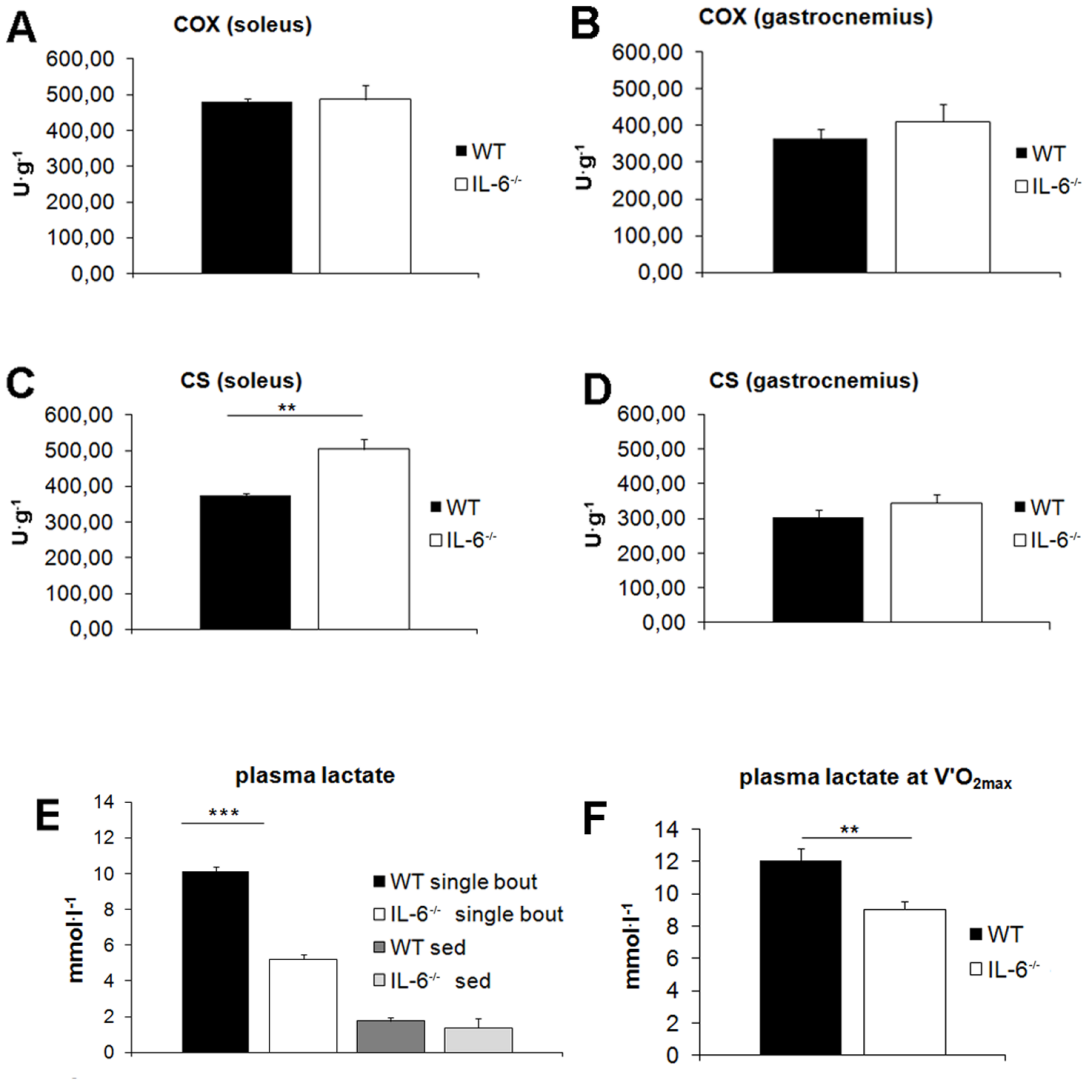
Post-exercise plasma lactate and COX, CS activities in skeletal muscles in WT and IL-6^−/−^ mice. COX and CS activities in *soleus* (A, C) and *gastrocnemius* (B, D) skeletal muscles were measured in lysates from non-exercising WT control mice and IL-6^−/−^ mice. Post-exercise plasma lactate concentration (E) was assessed both in non-exercising mice (WT at rest and IL-6^−/−^ at rest) as well as in animals subjected to single bout of exercise (1-hour run at 8 m^.^min^−1^). (F) Plasma lactate concentration at V′O_2max_ during maximal incremental running test until exhaustion in WT mice and IL-6^−/−^ mice. Data are presented as the mean ± SEM and symbols * denote values significantly different: **(P<0.01), ***(P<0.001). Statistical analysis was performed in GraphPad Prism5 (two-sided T-test, n = 7–11).

Because energy for endurance exercise is mainly provided by oxidative phosphorylation, we measured COX and CS activities in *soleus* (oxidative) and *gastrocnemius* (predominantly glycolytic) skeletal muscles of IL-6^−/−^ and WT control mice. There was no significant difference in COX activity in *soleus* (oxidative) muscle between IL-6^−/−^ and WT controls (Figures 4A). Interestingly, in this muscle we found that CS activity was significantly higher for IL-6^−/−^ mice (504±29 U^.^g^−1^, n = 10) than for WT animals (374±6 U.g^−1^, P = 0.002, n = 8), (Figure 1C). For predominantly glycolytic *gastrocnemius*, there was no difference in COX and CS activities between IL-6 knockouts and WT controls (Figures 4B, 4D) (probably because we did not distinguish between the red (oxidative) and white (glycolytic) parts of this muscle).

Since also anaerobic (glycolytic) pathways can provide working skeletal muscles with energy with lactate as a by-product, we measured post-exercise plasma lactate concentration in IL-6^−/−^ and WT mice subjected to a single bout of submaximal exercise and found it lower by about 50% in IL-6^−/−^ (5.22±0.26 mmol^.^l^−1^) when compared with WT control mice (10.13±0.25 mmol^.^l^−1^), (n = 7–9, P<0.001) (Figure 4E). Moreover, maximal plasma lactate concentration determined after the maximal incremental running test in the IL6^−/−^ mice amounting to 9.03±1.79 mmol ^.^ l^−1^ was significantly (P<0.01) lower in the WT mice (amounting to 12.03±2.27 mmol ^.^ l^−1^) (Figure 4F).

### Differences in skeletal muscle protein profile between IL-6^+/+^ and IL-6^−/−^ mice

**Figure 5:**
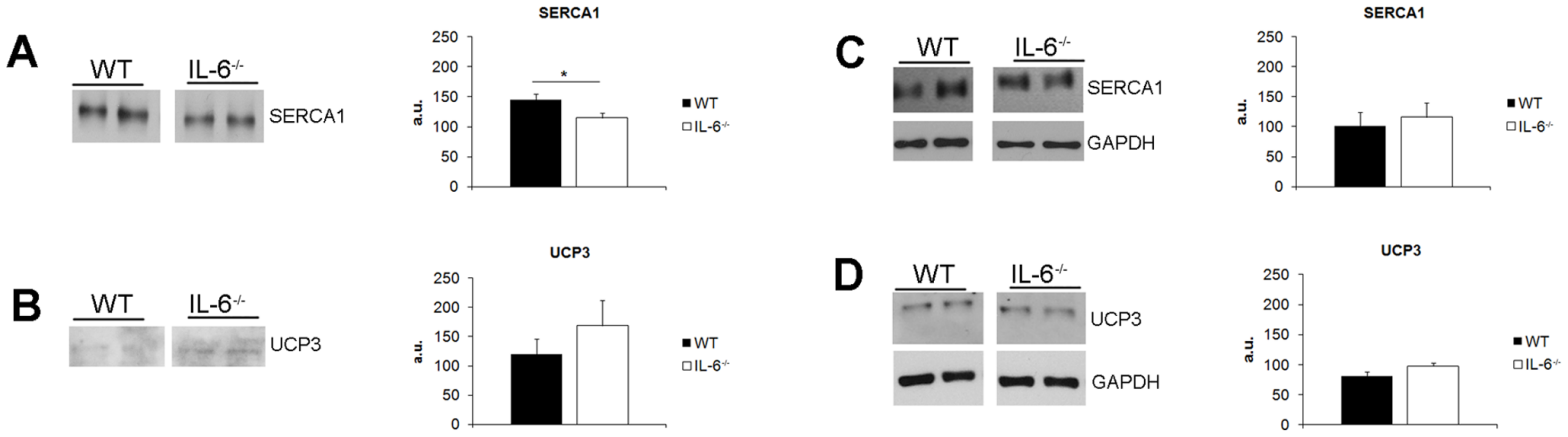
Skeletal muscle protein profile of WT and IL-6^−/−^ mice. Whole-muscle *soleus* (A,B) and *gastrocnemius* (C,D) lysates were used to assess the levels of sarcoplasmic reticulum Ca^2+^-ATPase 1 (SERCA1) and uncoupling protein 3 (UCP3). Values on the graphs represent means ± SEM. Representative Western Blot images acquired for the same membrane at the same exposure time are shown to compare protein levels between WT and IL-6^−/−^ mice with GAPDH used as the loading control for *gastrocnemius*. For *soleus*, we used Coomassie blue staining of protein gels as a loading control (not shown). The symbol * denotes values significantly different from WT controls: *(P<0.05). Statistical analysis was performed in GraphPad Prism5 (two-sided T-test, n = 7–8).

As described above, IL-6^−/−^ mice displayed increased CS but not COX activities in oxidative *soleus* muscle in comparison with WT control animals (Figures 4A and 4C). Moreover, IL-6^−/−^ mice accumulated much less lactate in plasma during single-bout exercise which might be due to lower muscle lactate production (as described in *Discussion* section). These observations may indicate energy deficiency in skeletal muscles of IL-6^−/−^ mice which would trigger compensatory mechanisms leading to reduction of energy expenditure. Indeed, in *soleus* muscle of IL-6^−/−^ mice we observed decreased level of sarcoplasmic reticulum Ca^2+^-ATPase 1 (SERCA1) in IL-6^−/−^ (115.1±8.4 a.u.) as compared to WT mice (145.3±9.3 a.u., n = 7; P = 0.04) (Fig. 5A), that could indicate reduction of energy expenditure whereas the level of UCP3 (participating in energy dissipation) was not altered (Fig. 5B). In predominantly glycolytic *gastrocnemius* we did not detect any changes in SERCA1 and UCP3 levels (Figures 5C and 5D).

## Discussion

We designed this study to elucidate the mechanisms of reduced endurance exercise capacity of IL-6^−/−^ mice reported earlier [7]–[9]. The main and original finding of this study is that our 10-month old IL-6^−/−^ mice displayed reduced exercise performance as evidenced by their lower maximal running speed (by about 23%) obtained during maximal incremental test (Figure 1A) and compromised endurance time while running on the treadmill at running velocity of 12 m^.^min^−1^ (Figure 2B), despite no difference in V′O_2max_ when compared to WT mice. Therefore, poorer running capacity observed in these mice cannot be explained by lower V′O_2max_, which in IL-6^−/−^ mice was similar as in WT mice (Figure 1D). It should be noted that in our study the impairment of running capacity was evident only at high running velocities, whereas during 2 h run at lower running velocities (v = 10 m^.^min^−1^), no difference in running performance between IL-6^−/−^ and WT mice was observed. These results are in agreement with the finding by Benrick *et al.* [11] showing no difference in endurance capacity between male IL-6^−/−^ and WT mice assessed during 50 minutes run at 75% of their V′O_2max_. Taking into consideration the key role of V′O_2max_ in determining endurance exercise capacity, our results indicate that IL-6 cannot be regarded as a major regulator of exercise capacity but rather as a modulator of endurance performance.

It has been postulated that IL-6 might affect exercise tolerance by its effect on AMP-activated protein kinase (AMPK) which is considered as an energy sensor of the cell by monitoring the AMP and ADP concentrations [16] and, thus, seems to play an important role in maintaining energy homeostasis during exercise (for review see [17]). It was demonstrated that elevated IL-6 levels during exercise [4] positively correlated with an increase in AMPK protein kinase activity [18] and, moreover, that AMPK could be activated by increased levels of IL-6 [18], [19]. On the contrary, lack of IL-6 resulted in diminished AMPK activity both in sedentary and exercised IL-6^−/−^ mice [20]. Since it was shown that during a single bout of exercise AMPK activation correlates positively with eNOS phosphorylation in the mouse aorta [21] and that AMPK co-immunoprecipitates with eNOS [22], we hypothesized that impaired exercise capacity of IL-6^−/−^ mice could be related to endothelial dysfunction and compromised NO-dependent peripheral vasodilation that is a limiting factor of tissue perfusion during exercise. Surprisingly, measurement of Ach-induced vasodilation of pre-constricted aortic rings revealed no impairment of peripheral endothelial function in IL-6^−/−^ mice (Figure 3G) excluding the presence of endothelial dysfunction in IL-6^−/−^ mice that could predispose them to impaired exercise performance. Maintenance of endothelial function in IL-6^−/−^ in the present study was also evidenced by the levels of nitrosyl-hemoglobin (NOHb) in red blood cells (reflecting NO availability in the circulation) which were no different from WT control mice (unpublished data). These findings stay in line with recent work of Schrader et al. [23] suggesting that IL-6 deficiency is not linked to endothelial dysfunction but, on the contrary, protects against angiotensin II-induced impairment of NO-dependent vasodilation.

An interesting finding of the present study was that the IL-6^−/−^ mice during running at sub-maximal velocities consumed systematically less oxygen than the WT mice (Figure 1G). This phenomena, *i.e.* lower oxygen uptake in IL-6^−/−^ mice compared to WT mice during running at the same absolute running velocity was originally reported by Fäldt *et al.* [7] (see Figure 4 therein). However, these authors postulated that reduced oxygen consumption during exercise causes a progressive oxygen depletion in these animals, which impairs their ability to continue running [7]. Moreover, Fäldt *et al.* (2004) speculated that impaired heart function and reduced capillarisation in the IL-6^−/−^ mice could be responsible for their poorer running capacity. In turn, our results suggest that the poorer running capacity reported in the IL-6^−/−^ mice was not caused by limited oxygen delivery. We have found no differences in the V′O_2max_ between the IL-6^−/−^ and the WT mice (see Figure 1D), indicating that the oxygen delivery to the working muscles as well as its utilization during exercise was well preserved in the IL-6^−/−^ mice. Furthermore, lower V′O_2_ observed during subsequent steps of the incremental test (see Figure 1E) and during running at 12 m^.^min^−1^ (Figure 1F) was not due to limitation in V′O_2max_ in the IL-6^−/−^ mice. Therefore, we strongly suggest that lower oxygen uptake observed during sub-maximal running in IL-6^−/−^ is not a sign of a limited oxygen delivery as suggested previously [7] but is related to the adaptive mechanisms aimed to achieve enhanced mechanical efficiency in IL-6^−/−^ mice.

In order to understand the physiological background of this mechanism, we measured some muscle proteins expression/activities involved in oxygen cost of work including sarcoplasmic reticulum Ca^2+^-ATPase1 (SERCA1), uncoupling protein-3 (UCP-3) as well as COX and CS activities (for overview see [24]–[26]). We have found lower expression of SERCA1 in *soleus* muscle in IL-6^−/−^ than in WT mice (Figure 5A). This could at least partly explain the lower oxygen cost of running at sub-maximal running velocities in the IL-6^−/−^ mice (for overview see [24]–[26]). Lower oxygen cost of the run in the IL-6^−/−^ mice could be also due to lower expression of UCP-3 in their locomotor muscles and lower energy dissipation during exercise (see e.g. [27]). However, in the present study we found no difference between UCP-3 expression in the *gastrocnemius muscle* of the IL-6^−/−^ mice when compared to the WT mice (see Figure 5D). Accordingly, no change in basal and post-running temperature was observed in IL-6^−/−^ mice when compared to the WT (see Figures 3C and 3D). On the other hand, we found higher citrate synthase (CS) activity (considered as a marker of mitochondria volume density - see *e.g.* [27], [28]) in the *soleus* muscle of the IL-6^−/−^ mice when compared to the WT mice (Figure 4C). However, the maximal COX activity measured in the *soleus* muscle of IL-6^−/−^ mice was not different when compared to WT mice (see Figure 4A). Moreover, the maximal COX activities measured in the *gastrocnemious* muscle of IL-6^−/−^ mice and WT mice were not significantly different (see Figure 4B). This suggest that the oxidative phosphorylation activity of the *soleus* and the *gastrocnemius* muscles in IL-6^−/−^ mice and WT mice are similar. Therefore, the lower oxygen cost of running observed in IL-6^−/−^ mice cannot be explained by higher oxidative phosphorylation activity in their locomotor muscles.

In contrast to humans, mice can successfully perform prolonged endurance running close to their V′O_2max_ (as observed during the measurements of V′O_2max_ in this study). This suggests that mouse locomotor muscles possess greater abilities to tolerate metabolic acidosis during endurance running. Little, however, is known about the role of IL-6 in developing of metabolic acidosis during prolonged high intensity running in mice. In the present work we demonstrated that plasma lactate concentration measured immediately after 1 h run at 8 m^.^min^−1^ was significantly lower (by out 50% i.e. 5.2±0.3 vs. 10.1±0.3 mmol^.^l^−1^) in IL-6^−/−^ than in WT mice. This is an original new finding showing lower lactatemia in the IL-6-deficient mice while performing endurance run at the same absolute running velocity (see, Figure 4E). This was accompanied by lower oxygen cost of running at submaximal running velocities in those mice (Figure 1G). Assuming that higher plasma lactate concentration is accompanied by greater muscle acidosis and lager disturbances in muscle metabolic stability resulting in lower muscle mechanical efficiency (see [24], [25], [29]), the observed lower oxygen cost of running in the IL-6^−/−^ mice could be related to lesser disturbances in muscle metabolic stability in their muscles during running at submaximal velocities when compared to the WT mice. It should be noticed that during this run (running at the same absolute running velocity amounting to 8 m^.^min^−1^) the relative exercise intensity in case of IL-6^−/−^ mice was much higher than in case of WT mice due to above mentioned lower maximal running capacity (by about 25%) found in IL-6^−/−^ mice (see Figure 1A) but still plasma lactate concentration in IL-6^−/−^ mice after this run was much lower than in WT mice. This is, indeed, an interesting new finding showing that IL6^−/−^ mice, despite of running at relatively higher running velocity, paradoxically accumulate much less lactate in the blood than WT mice. Interestingly, in the present study we have also demonstrated to our knowledge for the first time that the maximal plasma lactate concentration after fatiguing run to exhaustion in IL-6^−/−^ mice was significantly lower than in WT mice (see Figure 4F). Results collected in this study unable us to explain the reason for lesser accumulation of plasma lactate in IL-6^−/−^ mice as compared to WT mice since lower plasma lactate concentration in this case could be due to lower muscle lactate production and/or faster lactate uptake by various cells (see e.g. [30], [31]). Nevertheless, our results clearly indicate that IL-6 is strongly involved in the mechanism responsible for plasma lactate accumulation during exercise both during sub-maximal and maximal running velocities. In physiological conditions higher plasma lactate concentration at a given power output is associated with poorer performance of sustained exercise (see e.g. [32]–[34]) and, therefore, lower plasma lactate concentration in IL-6^−/−^ mice should be associated with better tolerance of sustained exercise. This could explain the preserved good endurance capacity of the IL-6^−/−^ mice during low intensity running when compared to WT mice (see Figure 2A). On the other hand, low plasma lactate concentration observed in IL-6^−/−^ mice during run at 8 m^.^min^−1^ when compared to WT mice could result from impaired anaerobic glycolysis as well as from faster glycogen depletion during exercise. Both these factors could be responsible for poorer running performance at high velocities found in this study in IL-6^−/−^ mice when compared to WT mice (see Figures 1C and 2B). Regarding the maximal incremental exercise, the lower plasma lactate concentrations found after this fatiguing run in IL-6^−/−^ mice when compared to WT mice (see Figure 4F) suggest that IL6^−/−^ mice possess poorer anaerobic glycolytic energy capacity than WT mice. This could at least partly explain their poorer maximal running capacity (near V′O_2max_). Interestingly, it was recently reported that poorer endurance swimming capacity of IL-6^−/−^ mice was accompanied by greater intramuscular glycogen depletion after fatiguing exercise [8]. This is in accordance with the findings by Kelly et al. (2009) [35] that IL-6 increases substrate availability within the muscle cell by increasing glycogenolysis and lipolysis.

In the present study, we found no difference in glucose tolerance between IL-6^−/−^ mice and WT mice, which indicates that, at least at rest, lack of IL-6 has no effect on glucose homeostasis. Our results are in accordance with the findings by [36] that IL-6 is not necessary for glucose production during non-exhaustive exercise. It was also recently reported, in contrast to previous observations, that IL-6 release in humans during exercise was not directly correlated with the release or uptake of exogenous substrate, nor to muscle glycogen utilization [37]. This suggests that more studies are needed to establish the quantitative significance of IL-6 in the carbohydrate metabolism during exercise in humans.

## Conclusions

The present study confirmed the previous reports showing that IL-6^−/−^ mice displayed reduced exercise performance as evidenced by lower maximal running velocity during maximal incremental test and shorter time to exhaustion at running velocity of 12 m^.^min^−1^. We provided novel evidence suggesting that poorer running capacity in IL-6^−/−^ mice is not due to lower V′O_2max_, impairment of peripheral endothelial function, glucose intolerance or impaired muscle oxidative capacity. Therefore, our results indicate that IL-6 cannot be regarded as a major regulator of exercise capacity but rather as a modulator of high intensity endurance performance. We also identified that IL-6^−/−^ mice displayed lower cost of running at a given sub-maximal running velocity linked to the lower expression of SERCA1 in *soleus* muscle and lesser plasma lactate accumulation during running at sub-maximal and at maximal velocities. This response seems to constitute an important compensatory mechanism limiting reduced exercise performance in IL-6^−/−^ mice.
